# Book Reviews

**Published:** 1817-09

**Authors:** 


### CRITICAL ANALYSIS
### *OF RECENT PUBLICATIONS,*
IN THE
### DIFFERENT BRANCHES OF PHYSIC, SURGERY, AND MEDICAL PHILOSOPHY.

---

*A Physiological System of Nosology, with a corrected and simplified Nomenclature.* By JOHN MASON GOOD, F.R.S. &c. &c. Dedicated (by permission) to the Royal College of Physicians. 8vo. pp. 566. Cox and Co. 1817.

IN a preliminary dissertation, we are informed that the plan of the work was laid down as early as the year 1808,* thus exactly according with the precept of Horace—*Nonum prematur in annum;* and, indeed, when we consider the length and labour of the work, we are almost inclined to wonder how a practitioner, engaged in professional duties, and other literary pursuits, could accomplish it so soon.

Having so often expressed our dissatisfaction at all the nosological attempts hitherto made, we had determined to impose a peculiar restraint on ourselves in the present instance, lest we should be warped either by prejudice or petulance. With this view we have paid the closest attention to the work; and shall, as often as possible, leave the author to speak for himself, and the reader to draw his own inferences.

"The main object (says Mr. Good in his preliminary Dissertation) of the present attempt is not so much to interfere with any existing system of NOSOLOGY as to fill up a niche that still seems unoccupied in the great gallery of physiological study. It is that, if it could be accomplished, of connecting the science of diseases more closely with the sister branches of natural knowledge; of giving it a more assimilated and family character; a more obvious and intelligible classification; an arrangement more simple in its principle, but more comprehensive in its compass; of correcting its nomenclature, where correction is called for, and can be accomplished without coercion; of following its distinctive terms as well upwards to their original sources, as downwards to their synonyms in the chief languages of the present day; and thus, not merely of producing a manual for the student, or a text-book for the lecturer, but a book that may stand on the same shelf with, and form a sort of appendix

---

* In the Transactions of the Medical Society of London. See our Journal, vol. xxv. p. 56.

to, to, our most popular systems of NATURAL HISTORY; and may at the same time be perused by the classical scholar without disgust at that barbarous jargon, with which the language of medicine is so perpetually tesselated; and which every one has complained of for ages, though no one has hitherto endeavoured to remedy it.

" The present, however, is but an attempt towards what is wanted, and is only offered in this view. How far such an attempt may be worth encouraging, and by what means it may be conducted towards a desirable degree of perfection, may perhaps be best determined by a brief glance at the chief nosological systems of the day, the nomenclature in actual use, and the general nature of the improvement proposed in the ensuing volume. It is the aim of this introduction to offer a few hints upon each of these subjects."

A section follows on " Nosological Systems." These, the author remarks, have been alphabetical, if such a modification is worthy of the name. " Another modification which has been had recourse to is that of the duration of diseases, as divided into chronic and acute. It is (continues Mr. G.) a modification of great antiquity, having descended to us from Aretæus and Cælius Aurelianus."

" A third modification has consisted in taking the *anatomy* of the animal frame as a ground-work for divisions; and consequently in assorting diseases, as has been done by Johnston, Sennert, and Morgagni, and since been recommended by Dr. Mead in his Medical Precepts and Cautions, into those of the head, chest, belly, limbs, and almost every other part. A fourth invention has fixed upon the supposed causes of diseases as a basis of distribution, and to this has been applied the epithet *etiological*, from the Greek term *αιτία*, a *cause;* it has acquired more popularity than any of the preceding, and was especially embraced by the schools of Boerhaave, Riverius, and Hoffman. Sometimes a mixt modification has been attempted, as in the nosology of Dr. Macbride, who takes *extent* for his first two general divisions of diseases, as being universal or local, *sex* for his third, and the age of *infancy* for his fourth and last. And sometimes, and far more generally of late years, the nosological system has been built upon the DISTINCTIVE SYMPTOMS of diseases—the peculiar marks by which they identify themselves, and, so to speak, become individualized: and such is the principle adopted by Sauvages, Linneus, Cullen, and all the most celebrated nosologists of recent times."

Having given this general historical view of nosology, Mr. Good enters into a more minute account of each writer. Plater he calls the morning star of symptomatology, as Serveto was of the circulation. Sydenham is said to have kept Plater always in view; and the attention of Sauvage to Sydenham first produced the attempt at reducing diseases to classes and orders. A general account of the *methodical* nosology follows, which is considered much too diffuse; but, but, on account of its intrinsic merit, Mr. G. advises every one to read it. The success of Sauvage, or rather the admiration of his work, produced many imitators. These are enumerated, and afterwards the names of some authors, who, without pretending to give their works the character of nosologies, offered only an arrangement of one department, which they had pursued, or as making part of some larger work.

The next general nosologist in order is Linnæus, in whose system Mr. Good remarks the changes from Sauvage. Vogel's is next examined, as an attempt to supply the defects in Sauvage's system—Sagar, as in some measure restoring the same.

"Upon the whole (says Mr. Good) it does not appear that the Nosologia Methodica of the Montpellier professor royal was much benefited, either in its arrangement or its substance, by any of these three attempts at improvement; while, in various respects, it was, perhaps, rendered less commodious and useful.

"VII. Such was unquestionably the opinion of Dr. Cullen, with respect to the two former of these—for that of Sagar was not then before the public—when he first thought of essaying his own powers in the field of symptomatic nosology; and hence, notwithstanding the later models that were before him, he resolved upon once more taking for a basis the original exemplar.

"The first objection, however, to this exemplar, which he seems to have felt, was not the mere series, but the nature of its classification. The main object he proposed to himself, and a more important he could not lay down, was that of brevity and simplicity; and the Sauvagesian classification offended in both respects. He determined, therefore, upon changing it, and re-casting the system from its commencement. Instead of ten classes, he conceived that four alone might suffice, formed, as he proposed to form them, of a caliber capacious enough to swallow up all the rest. He moulded his four classes accordingly, and distinguished them by the names of

I. Pyrexiæ, III. Cachexiæ,

II. Neuroses, IV. Locales:

and, influenced throughout the whole of his reform by the same spirit of simplicity and concentration, he reduced the forty-four orders of Sauvages to twenty, and his three hundred and fifteen genera to one hundred and fifty-one. He next carried his pruning-hook into the field of species: some he found to be repetitions of the same disease occurring under different genera, and others mere symptoms of other disorders, instead of distinct or idiopathic affections; all which were steadily lopped off; and in this manner the reduction in the species bore an equal proportion to that in the genera. The genera and species that remained were next enlisted into his own service, mostly with the respective names assigned them by Sauvages, though the definitions were generally re-composed,

 and and apparently modelled in consonance with the reformer's own practical observations.

"Thus completed, and fit for use, the new system was first started in the largest medical school of Europe, its author presiding at the head of it. It is not, therefore, surprising that it should instantly have rushed into popularity, and become a subject of general approbation. Yet it did not stand in need of this adventitious support to introduce it to public favour. Its aim at simplicity, as well in extent as in arrangement, was noble, and bespoke correct views, and a comprehensive mind; it promised a desirable facility to the student, and a chaste finish to the architecture of the nosological temple. The author showed evidently that he had laboured his attempt in no ordinary degree; and many of his definitions discovered a mastery that had never before been exemplified—pictures painted to the life, and of proper dimensions.

"To this extent of praise Dr. Cullen's system is fairly entitled,—an extent which ought ever to be borne in mind amidst the numerous, and in many instances exaggerated, exposures of its defects which have lately been exhibited, and which it seems to be a growing fashion to detail both at home and abroad; more especially in Germany, where it has been asserted, *ex cathedrâ*, and believed by extensive audiences, that, after all his pretensions, Cullen has done little or nothing for the improvement of nosology.

"That the system, nevertheless, has faults, and insurmountable ones, it would be absurd to deny; for they meet us at the very outset, and run through the whole of its texture and constitution. It is sufficient to notice the three following:—1. Defective arrangement. 2. Want of discrimination between genera and species. 3. Looseness of distinctive character in the last general division."

Several pages follow remarking the advantages and disadvantages of the Cullenian nosology, in which we could gladly have spared the allusions to the boundaries attempted by the late ruler of France, as well as a comparison of Cullen's *locales* to the cryptogamia of botanists; and still more his quotation from the Inferno of Dante, in illustration of diseases for which Cullen could find no place in either of his divisions. That we may not, however, do injustice to our author, we shall give the following specimen of his mode of enlivening a dull subject by an occasional sally of humour.

"Dr. Cullen, however, it must be admitted, has been as ingenious as he could; and contrived the means of giving, throughout all his classes, an entrance to diseases that have very little claim to admission. But the consequence is, that they make a sad medley, and, in many cases, have not the slightest affinity or family resemblance; of which we have a striking example in *psora* and *fractura*, which follow in immediate succession in the class of local disorders. Psora *(itch)* can scarcely be called a local affection, unless the term be appropriated appropriated to the skin generally, as distinguished from all the other parts of the frame; but, in this case, *trichosis* and *lepra* should have been placed in the same class, instead of in that of cachexies; while fractura could have no pretensions to such a class unless when compound. But it must certainly puzzle the best medical scholar in Europe, who is not acquainted with Dr. Cullen's arrangement, to discover the least connexion between *itch* and *broken bones,* and especially such a connexion as not only to draw them into the same class, but to make them immediate neighbours in the same order. Dr. Cullen, however, has ascertained that they are both *local* disorders, which entitles them to a common class, and both *dialytic* disorders, or produced by a division of continuity, which entitles them to a common order: and hence to the question, 'why is the itch like a broken bone?'—the student's answer is, 'because it is a dialysis:' an answer somewhat wanting, perhaps, in professional gravity, but the only one that can be given. And here it is probable we must stop; for there seems no possibility of advancing farther, and assigning any reason for the very close intimacy allotted to psora and fractura by fixing them in immediate succession. Yet there is, perhaps, quite as much difficulty in determining what could be the author's motive for placing *nostalgia* in any part of the same class.

"2. It is impossible to take a survey, however brief, of Dr. Cullen's system, and not to notice his very extraordinary confusion of genera and species. And the author is the more induced to advert to it, because, extraordinary as such a confusion must appear to all who are acquainted with the difference, Dr. Cullen is by no means the only nosologist of our own day who has run into the same mistake, as will easily be perceived before the close of this dissertation."

Some very fair remarks follow on the impropriety of making single diseases so many genera. "A genus," says Mr. G. "is a mere abstract term, a nonentity in nature, but highly useful,"—meaning, we presume, for artificial arrangement. Many other errors and inconsistencies are pointed out; and, even in the very best part of the Cullenian system, many inaccuracies are detected.

The writers on general or universal nosology being thus dismissed, a few remarks suffice for those who have made the attempt on a more confined scale. Selle's Methodical Pyretology is shewn to be very imperfect. Ploucquet's System, we are told, "is by far too complicated, and certainly not without its *nebulosity,*"—singularly distinguished by the author's fondness for long *crabbed words.* Pinel's Philosophical Nosology is "too refined for popular use, and too indistinct for practical benefit;" but his "arrangements of mental alienation" have been found very useful. To the other parts of his work many objections are added, which we conceive it unnecessary to notice.

Next

Next follows a view of the authors who have attempted to improve on the Cullenian system. Macbride has great credit "for a nice skill in the arrangement of his genera and species, in which there is a neatness and simplicity of which the author has endeavoured to avail himself, wherever the structure of his own system would allow, and which he has often left with regret when it would not."

Dr. Chrichton is next produced, and Mr. G.'s objections stated, excepting to the part which relates to mental derangement, and even that is not without its faults.

Dr. Darwin's system is commented on with much pleasantry; and, as we shall have occasion to mention it again, we are glad of an apology for making a further transcript.

"But (says Mr. Good) the direct death-warrant of the system consists in his making every single proximate effect (in common language, proximate cause or symptom) a distinct disease; for, as the same proximate effect or symptom may be produced by several, or by each of what Darwin calls proximate causes, and which constitute his classes, it follows that the very same species or specific disease must, in such cases, belong equally to some order or other of several, or of all the classes of his system. And such, to the student's embarrassment and surprise, he will find, upon examination, to be the real fact. Thus, while *variola* (small-pox) is arranged under cl. II. ord. I. gen. iii., *eruptio variolæ* (small-pox eruption) occurs under cl. IV. ord. I. gen. ii. So *hydrophobia* appears first in I. III. i. and afterwards in III. I. i.; *diabetes* in I. III. ii. and again in IV. III. i.; *palpitation of the heart* in I. II. i. and again in I. III. iii. being twice in the same class: and of so many others."

The author next noticed is Dr. Parr, of whose Dictionary we should say, Mr. Good speaks in terms of exaggerated praise, were it not for a sentence so just as at once to convince us what we are to expect from such a medical writer.

"He [Dr. Parr] was his [Mr. Good's] colleague in conducting, for some years, two of the most extensive literary works of the present day, though not the Dictionary in question; and he can affirm, from a full knowledge of his talents, that he was a man of deep study, comprehensive capacity, and extensive learning. His mind, indeed, was so widely fraught with miscellaneous information, that few subjects could come amiss to him. His Dictionary gives evident proof of his having been alive to every novelty in his own profession, and of his readiness to allow its merits. He was far more disposed, indeed, to be satisfied with the opinions of others than with those of himself; and chiefly failed in a want of deference to his own judgment. In laying down the outline of his system of diseases, which he only attempted upon a full conviction that a work of this kind was extremely wanted in the medical republic, he had his eye chiefly directed to the nosological method of Selle, and the botanical method of Jussieu. It follows, therefore, that his primary division would would consist, not of *classes*, but of what he intended to be *natural orders* or *families*. These orders are twelve, whose names are taken from the classes or orders of Sauvages or Cullen, with the exception of one, SUPPRESSORII, which is borrowed from Linnéus.

"Here again, therefore, we have a great and noble aim, whatever be the success of its accomplishment. But, as a natural system, even in botany, is to the present hour, and perhaps always will be, a theoretical rather than a practical idea, there seems very little expectation that it can ever be realised in medicine."

At present we shall only remark, that we do not consider "a mind widely fraught with *miscellaneous* information," or "the conductor of two of the most *extensive* literary works," as expressions complimentary to a physician or a medical writer.

Lastly, Dr. Young's "Introduction to Medical Literature, including a System of Practical Nosology," is, in our opinion, very fairly estimated. "Though limited to a single 8vo. it comprehends a complete course of medicine, and directs the student to the best authorities and sources of information; in this respect answering the purpose of Plouquet's seven 4to. vols. with a great saving of expense, a prodigious saving of time, and by a far nearer and pleasanter pathway."* It is not to be wondered if even Dr. Young failed, like his predecessors, in his attempts at nosology. His plan, however, comes the nearest to Mr. Good's, though essentially distinct. We ought to add that Dr. Young published in 1813; and it has already been remarked that Mr. Good's work has been nine years on his table. Whatever similarity, therefore, may be detected, must arise from a coincidence of opinion, and not from plagiarism.

This general review concludes with a slight notice of those nosologists who have confined their labours to "a single family or group of diseases." To the name of Selle, Pinel, and Crichton, are now added Plench, Willan, Abernethy, and Bateman. After a few remarks on the merits of each, the author commences his own plan by some observations on medical nomenclature: A considerable portion of this, he observes in a note, was published in the Memoirs of the Medical Society of London, and for which the author received the Fothergillian medal. There can be no difficulty in showing the absurdity of many of our terms, the erroneous

---

* We are glad of this opportunity of doing justice to Dr. Young's book. Our remarks on it at the time were confined to a certain passage, which, from the nature of the performance, seemed the most candid method of pointing out what the reader might expect from the whole.

opinions opinions on which they were originally formed, nor the propriety of changing them. But, in the latter, the difficulty is great. As we remarked before,* to alter the terms adopted by authors universally respected, and in well received works, is a violence that few will submit to. This difficulty may, however, be confined to the present generation, and be greatest in us who are arrived at an age when we are apt to become *laudatores temporis acti.* Besides this, we are forced to acknowledge, that, having found the study of medicine quite sufficiently laborious, and longer than we can expect our usefulness in life to continue, we have been always shy of encumbering ourselves with any addition to our troubles. What we have just said, will be considered as a further acknowledgment of the danger we feel lest prejudice should direct any of our remarks; and we again repeat the wish, that the reader, whilst he peruses us, would keep that danger in view. After this confession, we enter on the

"*Scope of the present Design.*

"I. It is obvious then (says Mr. G.) that the healing art stands in considerable need of improvement in its two important branches of NOSOLOGICAL ARRANGEMENT and NOMENCLATURE: and it is, among other points, to an improvement in these two branches that the ensuing pages are especially directed.

"In giving an outline of what the author proposes in order to accomplish this purpose, it is of little consequence which of these two divisions shall first pass in review before us: let us then begin with that of language or NOMENCLATURE, as being, perhaps, freshest in the memory.

"In the hope of giving some degree of improvement to the medical vocabulary, as far as he may have occasion to employ it, the author has endeavoured to guide himself by the following general rules. Firstly, a strict adherence to Greek and Latin terms alone. Secondly, a use of as few technical terms as possible, and consequently a forbearance from all synonyms. Thirdly, a simplification of terms, as far as it can be done without violence or affectation, both in their radical structure and composition. Fourthly, an individuality and precision of sense in their respective use."

In taking a view of the languages best suited for nomenclature, no scholar can fail to prefer the Greek, which by

* See London Med. and Phys. Journ. vol. xxv. p. 56. It may be right to remark, that the nomenclature is much improved since its former publication; and, in one instance, the author has attended to our hint, or, which is more probable, improved his terms by subsequent reflexion.

common consent has found its way into every branch of philosophy, in proportion as each has been better cultivated, and hence become more precise in its terms. Whenever it is found necessary to make compound terms, the Greek has so many advantages, that even the Germans, with a language scarcely less flexible, have not scrupled to submit to the general choice of all Europe. Mr. Good has also the advantage of early authorities, from the best writers, for most, if not all, his generic terms. In his specific terms, he is less scrupulous; but, even here, he rarely wanders beyond the Greek and Latin. The next object in philosophical language is *simplicity*. We shall copy the following paragraph as a specimen; it may come before us again, when considering the execution of the work, for, at present, the reader will recollect we are only engaged on the preliminary dissertation.

"2. The machinery of every art and science (says Mr. G.) becomes simpler, and its auxiliary powers fewer and less needed, as it advances towards perfection. It is the same with their technology. While we are but loosely acquainted with the principles of an art we speak of them with circumlocution, and employ more words than are necessary, because we have none that will come immediately to the point. As we grow more expert we learn to make a selection; we give to many of them a greater degree of force and precision; and separate those that are thus rendered of real value from the "leather and prunello," the heavy outside show of useless and unmeaning terms with which they are associated; and thus gain in time as well as in power. In unison with these ideas, the author, as soon as he has pitched upon a word that will best answer his purpose, will be found, as he hopes, to adhere to it wherever he has had occasion to advert to the same idea, without indulging in any play of synonyms, or different terms possessing the same or nearly the same meaning. Marisca and hæmorrhois have been equally employed by medical writers to distinguish the disease which we call vernacularly *piles*. The first is a Latin term, and refers to the tubercles of the disease, and the second a Greek, and refers to a discharge of blood which occasionally issues from them. As commonly used they are direct synonyms, notwithstanding this difference of radical meaning, and either might answer the purpose; the diversity of the disease being pointed out by distinctive adjuncts, as cæcà, mucosa, or cruenta. Sauvages and Sagar, however, have employed both; but have laboured to establish a difference, without having succeeded even in their own judgment. So that, in these writers, we have one and the same disease described under two distinct genera in distinct classes; the first occurring in Sauvages under class I. ord. V. entitled, VITIA, CYSTIDES: the other under class IV. ord. ii. entitled, FLUXUS, ALVIFLUXUS, and introduced with this remark, "HÆMORRHOIDES vero nihil aliud sunt quam MARISCÆ, gazæ apud Aristotelem."

In

In the present system, marisca* is alone retained; and the author has preferred it to hæmorrhois, first, because hæmorrhage is only a symptom that characterises a peculiar species, or rather, perhaps, a variety of the disease; and next, because hæmorrhois, or rather hæmorrhoidæ, (αἱμοῤῥοΐδαι) was employed among the Greeks, as well vulgarly as professionally, in a much wider sense than that of modern times, and imported flux of blood from the vagina, as well as from the anus; and, in fact, from any part of the body, when produced by congestion and consequent dilatation of the mouths of the bleeding vessels, which were supposed in every instance to be veins. So Celsus, "Tertium vitium est, ora venarum tanquam capitulis quibusdum surgentia quæ sæpe sanguinem fundunt: αἱμοῤῥοΐδας, Græci vocant. Idque etiam in ore vulvæ fæminarum incidere consuevit." To the same effect Hippocrates, Lib. *de Morb. Mulier.* Galen uses it in a still wider extent, *De Morbus Vulgaribus:*† and hence the woman with an issue of blood in St. Matthew, ch. ix. 20, is termed in the Greek text γυνὴ αἱμοῤῥοοῦσα.‡ Gaza (γαζα), the term used by Aristotle, would have answered as well as marisca, but that it is less common in the present day, and an exotic term even in the Greek. Hesychius calls it a Persian word, and Scaliger coincides with him; translating it, "thesaurus, reditus, tributus," "*a treasury*," or place of deposit or accumulation, chiefly of tribute or taxes. It is rather an Arabic than a Persian term, though both countries use it under different inflexions. The Arabic root is ——§ *(khazi)* 'a blush or ruddy flush,' whether from fulness, shame, or modesty; whence the verb —— *(khaza)* 'to produce blushes, erubescence, or suffusion;' and hence —— *(khazan)* in *Persian*, signifies 'autumn, or the season of fulness and erubescence;' while —— *(khazain)*

"* The term occurs in Juvenal, in its medical import, ii. 12.

podice levi
Cæduntur tumidæ, medico ridente, MARISCÆ.

"In Martial it occurs frequently in the literal sense of fici, 'fleshy or succulent figs or raisins.' The spongy and succulent bulrush of the marshes, or grounds overflowed by the sea, was called *mariscus*, from its habitation *à mari:* and hence, probably, the name of the spongy and succulent tubercles which constitute the piles. Our English *marsh* has the same origin as *mariscus*."

"† Comm. VI. cap. XXV."

"‡ Sauvages, not sufficiently attending to this extensive sense of the term among the Greek writers, represents this disease in St. Matthew as a *marisca cruenta*, or case of bleeding piles, instead of a catamenial hæmorrhage. 'Hæmorrhois, à Græce *aima* et *rheo* est fluxus sanguinis EX MARISCIS; unde mulier in Evangelio *hæmorrhoissa* dicta fuit.' Vol. I. p. 164. *Apud Mariscam*."

[§ The Arabic characters are in every place given by Mr. Good, but omitted in our review, as unnecessary for the oriental readers, and useless to others. Every one also knows the difficulty of procuring a correct copy of them.—EDIT.]

*zain)* in *Arabic*, is 'a garner, treasury, or repository for the fulness of the autumnal months;' literally *cella*, *cellula*, *gaza*, or *gazophylacia*, as explained by Hesychius."

The above passage may seem to contain more learning than is necessary; were this the only objection, we should readily excuse it, especially as it introduces us to a most important part of all science, and much less attended to in medicine than in any other. We mean those *επεα πτεροεντα*, the prefixes and affixes of the Greek language. If Mr. Good had done nothing more than call the attention of medical writers to precision in this single instance, we should admit the great services he had rendered us. The subject, and a few others to the same purpose, occupies more than twenty pages of close printing, which, though not at all too much, is more than we can, in justice to the author, transcribe or epitomize. It may, however, afford some gratification, when we assure him, that, after perusing it three times with increased attention, we have found every time fewer objections to make.

This division of the work concludes with an inquiry into the various senses in which authors have used several terms of frequent occurrence. Among these, we meet with asphyxia, hemerolopia, nuctolopia, asthesia, exanthema, pyrexia, accessio, insultus.—We wish the author had extended the number, and made his remarks still longer.

Having completed his observations on language, Mr. G. proceeds—

"A knowledge of the animal frame involves a deep and comprehensive acquaintance with three distinct branches of natural science; anatomy, by which we become acquainted with the structure of this frame; physiology, which teaches us its various functions; and *nosology or pathology*, which unfolds to us the diseases to which it is subject. Unfortunately each of these branches has hitherto been taught by a different, instead of by a common, method; and hence the student, instead of proceeding with each at one and the same time, and with a single expenditure of labour, is compelled to apply himself to every one separately, and by a kind of new and unconnected grammar."

"Having conceived the possibility of a nosological system, whose primary divisions should take a physiological range, and follow up the diseases of the animal fabric in the order in which the physiologist usually develops its organization and its functions, the author had next to determine at which end of the series he should begin; whether with Haller, at the first and simplest vestige of the living fibre, and pursue the growing ens through all its rising stages of evolution and elaboration to its maturity of figure and sensation; or, with the physiologists of later times, to take at once the animal frame in its mature and perfect state, and trace it, from some well-defined and prominent function, through all the rest; which, like links in a circular circular chain, may be said to issue from it, and to be dependent on its existence and properties.

"The author was soon led to a preference of the second scheme. It is by far the simpler of the two, and directly harmonizes with the fundamental principle, which runs through all the systems of zoology, botany, and mineralogy, of forming the arrangement and selecting the characters from the most perfect individuals as specimens. He decided, therefore, upon taking the more prominent functions of the human frame for his primary or classific division, and the more important of their respective organs for his secondary or ordinal; and without tying himself to a particular distribution of the former in any authorized or popular use at the present moment, to follow what appears to be the order of nature in her simplest and most intelligible march.

"To repair the exhaustion which is constantly taking place in every part of the body from the common wear and tear of life, it is necessary that the alimentary canal should be supplied with a due proportion of food, the procuration of which, therefore, constitutes, in savage as well as in civil society, the first concern of mankind. The food thus procured is introduced into a set of organs admirably devised for its reception; and its elaboration into a nutritive form constitutes what physiologists have denominated the DIGESTIVE FUNCTION. The diseases then to which this function is subject will be found to create the first class of the ensuing system."

The function of respiration follows; sanguification next. Then the brain and nervous system; "the sexual function;" "the excernment function," comprehending secretion.

"It will yet remain (continues Mr. Good) to create a class for EXTERNAL ACCIDENTS, and those ACCIDENTAL MISFORMATIONS which occasionally disfigure the fœtus in the womb. This will constitute the seventh; and under these seven classes it will possibly be found that all the long list of diseases may be included which man is called to suffer, or the art of medicine to provide for."

Some remarks follow to show that this order is strictly physiological, and has been that of the best authors of physiological systems. Several objections, however, are started against the best of these writers, viz. M. Vicq. d'Azyr, Richerand, and Bichât. We shall only remind our readers, that these men were not nosologists, but physiologists; it will, therefore, be sufficient, if we pursue Mr. Good's remarks, and only consider a reference to the above writers as, in our opinion, unnecessary. The same may be said of his difficulty in distinguishing genera from species, and species from varieties, which is illustrated by botanical and zoological allusions.

Mr. Good regrets that some difficulties still exist after defining a species to determine its genus, remarking that frambœsia is, by Cullen, placed among the impetigenes, but ought ought rather be among the exanthems. That Dr. Parr, of whose dictionary he speaks so highly, places *pestis* in the order of exanthemata, Cullen expresses his doubt; but Parr, in a subsequent part of his dictionary, declares, that his own arrangement is improper; that pestis ought to be reduced to a variety under the "asthenic remittents." Similar difficulties occur in chlorosis, but Mr. G. very justly observes, that the same difficulties occur in all the attempts at arranging the other productions of nature into any artificial classes. In all such cases, we are advised to wait for additional information, and the author conceives,

"It is not improbable that some future nosologist (should the present work have any pretensions to futurity) may be able to assign more correct places to several of the genera or species of the ensuing arrangement than those they now occupy. It should be well remembered, however, that the principle of this arrangement consists in determining the proper class of a disease from the *general function* that is injured, and not from any *particular organ*, which only regulates the subordinate divisions: and that, where two general functions are injured at the same time, that constitutes the class which appears to be most prominently affected. Thus scrofula and scabies, which seldom extend deeper than the secretory vessels of the skin, belong necessarily to the class ECCRITICA, or that comprising diseases of the EXCERNENT FUNCTION; while variola and rubeola, though equally occupying the surface, belong to the class HÆMATICA, or the diseases of the SANGUINEOUS FUNCTION; which, in both these cases, is primarily and chiefly affected, as is obvious from the pyrectic action of the heart and arteries. So, while gastritis and enteritis belong also, as inflammatory affections, to the HÆMATIC class, dyspepsy and cholera, though disorders of the same organs, must necessarily be referred to the class CŒLIACA, or that comprising the diseases of the DIGESTIVE FUNCTION, this being the part of the animal economy which is hereby chiefly or wholly disordered.

"It may, perhaps, be objected that this is to travel over the same region a second or even a third time. It is, however, always in pursuit of a different object. It is to follow up the family of diseases that appertain to a particular function; while, to avoid having our attention distracted, we leave every other function and the diseases belonging to it, untouched. We pursue the same plan in zoology, in botany, in mineralogy. In a common region we discover discrepant specimens and bring them home at different times; exploring it on one occasion for one purpose, and on other occasions for others: and we then separate and arrange the productions into different classes and orders for scientific study, notwithstanding that nature has produced them in a common quarter."

Mr. Good gives several cogent reasons for excluding *dolores* as a genus, or species, and for arranging them among the *symptoms* of those affections in which they usually occur.

Lastly,

Lastly, we are informed, that the vernacular synonymes are added in the three modern languages, English, German, and French. This the author calls a new attempt. In England, we believe it is so, in works strictly nosological. His technical synonyms are likewise derived from the three most extensive languages of antiquity—Greek, Latin, and Arabic. Occasionally, where the Arabic names are Persian, he has added the initials, or other marks, of these cognate tongues; and, in a few instances in which they are peculiarly expressive, he has also superadded Persian or Turkish names, even though different from the Arabic. At times, indeed, the Arabic writers themselves employ a Persian or a Syriac term, for many of them were Persians or Syrians by birth, &c. This leads the author to a consideration of oriental literature, the cultivation of which, he inforces with much eagerness, concluding, at last, with a recommendation of Galen and Celsus among the ancients for all that is contained in the writers antecedent to the Arabian Caliphats.

We have thus completed our abstract of the preliminary dissertation with as little interruption as possible, that we might not be suspected of lessening the interest the reader should feel, or of inducing him to form a premature judgment. We shall now proceed to offer our remarks on the passages as they have occurred.

Had Celsus, Aretæus, Boerhaave, Mead, or Sydenham, attempted unsuccessfully any thing like a nosological table, we might have admired the boldness of each of their successors in so arduous a task: *Quem si non tenuit magnis tamen excidit ausis.* But, what resemblance is there between the *arrangement* of some of these illustrious writers, and those systems of nosology which have sprung up since? Sydenham, it is true, expressed a forlorn wish, but he lived to witness the dreadful effects of such an attempt for that class of diseases, in the history and treatment of which he has left scarcely any thing unfinished * We perfectly agree with Mr. Good, that an alphabetical arrangement is not to be considered an attempt at a system of nosology. On the contrary, is it not a tacit acknowledgment in the author, that, for want of a better method, he has recourse to one which breaks every catenation of the various parts, and makes each article a distinct series of observations and instructions?

What Mr. Good calls the duration of diseases, is, indeed, a most important distinction, when explained by the terms

---

* Compare his preface with his remark afterwards, that the malignant fever has destroyed more than the sword.—Was this a prophecy of the influence of the word Typhus in our own days?

acute and chronic. This is the true natural division, and means no more than to distinguish between those diseases which require the immediate interference of the physician, and those which admit of deliberation. Happy were it for mankind, had that distinction been as constantly kept in view as it has been invariably enforced by the ablest writers. We are at a loss to conceive, what can be meant by taking "anatomy for the modification of nosology." None of the writers instanced attempted any thing like nosological systems, and Dr. Mead, who is described as bringing up the rear, expressly tells us, in the passage to which Mr Good refers, that the animal machine is not made by parts, but altogether; producing an authority,* to which Mr. Good is never backward in paying due respect. Neither Boerhaave, Riverius, nor Hoffman, attempted any nosology, and the etiological, which is palmed on them, is, in fevers at least, more chargable on the latest and most popular of all the nosological writers. In our opinion, every division of diseases, from the days of Aretæus to Sauvage, was rather an arrangement for convenience than founded on any attempt at a philosophic system. Even Sauvage could scarcely deserve any other character. His descriptions are so minute, his authorities so universal, and, in all, the therapeutic part is so generally added, that his work may be called, if not a dictionary, a *catalogue raisonnée*, arranged according to certain distinctions which he fancied more convenient than by the alphabet. The arrangement by DISTINCTIVE SYMPTOMS, Mr. Good very properly observes, is the only one which can be of any practical use. Symptoms by an accurate and well instructed observer may be ascertained, but the seat of a disease can often be only discovered when it is too late to apply a remedy. Mr. G. considers Sauvage's work as too diffuse, yet so useful, that the student who neglects to read it carefully through, neglects one of the most important parts of his education. We, on the contrary, though not less disposed to compliment that work, conceive that the student who reads it through carefully will totally mistake the author's intention, and rise with a mind darker than chaos and not less confused. This caution cannot, however, be necessary, because we do not believe there is a student in the world who could read it carefully through. As a book of reference, it is invaluable, and as such will be found in every well-furnished medical library. The same may be said of all the succeeding nosologies, till we arrive at Cullen.

---

In

In contemplating the character of this illustrious professor, we know not whether to admire most his genius, his industry, or that elegance of style, which has done much to render physic a popular study. We ought to add, that, notwithstanding the great injury we conceive the most important part of medicine has suffered by him, we are not at all disposed to undervalue his talents, or to doubt the goodness of his intentions. Never can we forget the modesty of that note, "*De Elephantiasi, Lepra, Frambœsia, et Trichomate,* UTPOTE MORBIS A MEIPSO NUNQUAM VISIS *amplius statuere non ausus sum.*" Had he extended the same remark to Camp and Tropical fevers, his work might have been as free from objections as such a work can be. But the great mischief that followed his system, arose from its becoming the text book for his lectures, and unfortunately many of his hearers, rendered arrogant from the just celebrity of their teacher, began and finished their medical education in a school which furnished so few sources of practical knowledge. They were taught, indeed, all that could be taught in an university; but too many considered, that in that school were unfolded all the arcana of physic. This is now so universally understood, that nothing more need be said on the subject. We shall, therefore, continue our remarks on the extracts we have made from Mr. Good.

Cullen, we are told, seeing the diffusiveness of his predecessor, attempted to include the whole in four classes. After pointing out the difficulties attending such an attempt, Mr. Good expresses his displeasure at what he conceives a too severe remark *ex cathedra* in Germany. Yet this advocate immediately afterwards admits "insurmountable faults" in the system of Cullen. Is not such a censure on such a science as medicine infinitely stronger than the remark from the German chair? We perfectly agree, however, with Mr. Good, and only regret that the state of medicine was such in Cullen's days as to force from him a Nosology in any form.

The successors of Dr. Cullen, who have attempted to improve him, have been, we are told, Mac Bride, Darwin, Parr, and Young. Of Mac Bride, Mr. Good has said enough; on Darwin, he has been pleasant enough, but such is the difference of taste, that we cannot help detaining our readers on the death-warrant, as Mr. Good is pleased to call it, of this system. What, let us ask, are the objects of nosology? To distinguish diseases, it may be answered. And to what purpose? That we may know how to treat an animal whose actions or external appearances are different from their ordinary state. Now, if the business of arrangement is to teach us how to act, the first question will be, whether we must commence our operations instantly, or whether we may be allowed time even to consult? And this makes the first grand division between acute and chronic diseases, in their attention to which, the ancients, without any nosological tables, so much exceeded the moderns.

" The symptoms of a disease, (says Mr. Good,) indeed, have not unfrequently been said to constitute the disease itself. This is not perhaps strictly true; they are rather an algebraical character designating an unknown quantity, but which, in the hands of a skilful mathematician, may be managed as readily in working a proposition as if such unknown quantity were a sensible object.

" It is hence that the writings of Hippocrates and of Sydenham are so highly and deservedly esteemed; and will be so as long as medicine shall be practised."

Some remarks follow on Celsus, whom we shall presently attend to, and we could wish Aretæus had been introduced also. Our first business, according to the instructions of these great men, and particularly the last named, is to discover symptoms which immediately endanger the life of our patient, or the destruction of some important organ. When these are relieved, we are called on for diagnosis and prognosis, and for these we require a most exact description of those external appearances which mark the disease, and of the particular character in each which usually designate a favourable or fatal issue. Hence it follows, that, as in the same disease the symptoms are different in different stages, an arrangement to be useful must be modelled accordingly; nor can we illustrate this better than by Mr. Good's objection to Darwin. If, when called to the bed-side of a person during the eruptive fever of small-pox, we discover synocha, are we to wait to see whether it will prove synochus, that is, *typhus versus finem*, or whether *tertio die incipit eruptio*, &c. Should the case prove highly inflammatory, would not our patient be irretrievably lost by such delay? If, on the other hand, the eruption has begun before we see our patient, are we not to direct our attention to the symptoms immediately before us; and is a disease with an eruption and one without an eruption to be discovered by the same characters? It may be urged, that it becomes us to make every enquiry. But this will lead us to etiology, the danger of attending to which in this state of things, we shall consider presently.

The great object then in acute diseases, is to enable ourselves to form a prompt decision, and it was not altogether erroneous that some of the ancients began their instructions by by an immediate application to *signa et agenda.* Celsus, who cannot be considered an original author, before he discriminates any diseases by name, marks certain symptoms or signs which require or authorize blood-letting, vomiting, purging, and other active remedies, which the urgency of the symptoms may imperiously demand, even though we are ignorant what the disease may ultimately prove. This then we consider the first and most important division of diseases, and so important, that in our opinion it should be unfettered with any distant considerations, which may paralyze our attempts at giving immediate relief, or preventing fatal consequences.

But, to come nearer to the point, typhus is called *febris contagiosa.* The *cause*, therefore, is contagion, and unhappily this etiological distinction was thought a sufficient argument in favour of a practice which every family in the kingdom has, at different times, had cause to lament.

Let us recollect, that the ancients had none of the advantages we derive from the examination of those who have died of these fevers. Even Sydenham was deficient in this respect. But, by their mode of judging from the immediate symptoms, they acted with decision; and experience has shown, that, by such decision, they escaped those disasters which dissection has at last taught us are often the result of our nosological cautions.

Thus, whatever may be said of Darwin, to us, it appears, that, if the object of nosology is to direct us in diagnosis and practice, there is the utmost propriety in dividing small-pox into all the orders and genera which its appearance in its different stages exhibits.

Respecting Dr. Parr, in our opinion, when coupled with Mr. Good, he has, to use the expression of the latter, given the death-warrant to all nosology. "As a natural system, (says Mr. Good) even in botany, is, to the present hour, and perhaps always will be, a theoretical rather than a practical idea, there seems very little expectation that it can ever be realized in *medicine.**" Now, without the necessity of a pun, the study of physic is the study of nature, and of all the branches of that study, medicine is the most important, and that which requires the most immediate reduction to practice; any thing, therefore, which takes off our attention from the immediate operations of nature is dangerous. If diseases were to be exhibited like fossils or plants, it would matter but little how artificial their arrangement might be,

* See above, page 216.

Chemical proportions, general configuration, or the form of certain parts at a certain period of existence or growth, may be sufficient for subjects on which we operate at our leisure, or which we turn to and produce as often as we wish to point out their distinguishing characters. Is such the object in designating acute diseases? At some very distant period, it may be accomplished in *chronic* cutaneous complaints, but, for this purpose, hospitals, sufficiently capacious, must be endowed, in which professors, sufficiently honest and enlightened, must preside.

This leads us to the consideration of those attempts of some authors to form a nosology of only certain "groups or families of diseases or monograms." Of these, Plenck, Willan, and Bateman, are principally noticed. The two latter have frequently come before us, we shall, therefore, only slightly mention the objections we find made to Willan, reserving our further remarks to the instances we shall produce from the system of nosology before us. Willan is said to be best suited for his restricted system, and that it would not well be interwoven into a larger plan.

"As it is, indeed, it stands in need of no small degree of modification to clothe it with all the perfection it deserves; for several of his orders would make better genera; almost all his genera are decided species, while his species are seldom more than varieties, and are in many cases so denominated by himself."

We confess ourselves not prepared to admire a part which will not adapt itself to a whole, nor a whole which will not harmonize with a well-constructed part.

At length we have arrived at Mr. Good's own plan, as stated in his preliminary dissertations. The general remarks on language have been already noticed. We now add, that it is with much pleasure we find no admission to *pseudos* and *oideses*, those disgraceful subterfuges of ignorance or indolence. We are aware such terms are to be met with in the methodical synopsis, or systems of other branches of natural history. But, besides what we have before remarked of the impropriety of attempting to confine nosology to the same artificial laws, we may add, that every science has divested itself of these terms in proportion as it has been more correctly taught. Ray, in his Synopsis Plantarum, omits four out of five *pseudos*, and Linnæus admits none. The latter is not less careful in discarding the *oideses*. Can there be a more wretched view of the most important science, than that, in the present day, it should avail itself of terms discarded by all the reformers in the other branches of natural philosophy.

After

After our decided approbation of Mr. Good's diligence, and, as far as our inquiries have extended, his accuracy in terminology, it is with no small concern, that, as soon as we enter on his arrangement, we meet with the words *nosology* and *pathology* used as synonymous. *Pathology* has always been applied to that knowledge by which, from the *sufferings* of the patient, we may detect the nature, if not the seat of his disease. Nosology is a modern science as well as a modern word, and, by the moderns, is applied to an artificial arrangement, by which we are to ascertain the order, class, genus, or species, of diseases.

We are absolutely frightened, when we re-peruse our own words, and still more so, when we recollect that we have been reading of "groups and even families of diseases," and that the arrangement or march of these is to vary according to the taste of every fresh nosologist. Can there be a stronger proof of the impracticability of accomplishing an object than that no two writers should agree, and that, in the midst of their attempts, they should lose sight of the only purpose to which every thing useful in the study of medicine is directed. Mr. Good informs us, that he has preferred according to the plan of modern physiologists, to take for his arrangement the animal frame in its more mature and perfect state, and trace it from some well defined and prominent function. He then speaks of links in a chain, and urges the simplicity of his plan as according with all the systems of zoology, botany, and mineralogy, forming the arrangement, and selecting the characters from the most perfect individuals as specimens. Is it possible, this learned and ingenious writer, after nine years' consideration, is not aware that this very sentence renders his whole labours useless or something worse. In every acute disease, are we to wait for the maturity and perfection of our specimen?—Certainly not; but, it may be answered, by accurately ascertaining the true character from such a specimen in its most mature and perfect state, we ascertain the true disease, and, having once done so, it will not be difficult to mark the varieties. Can we wish, let us ask, for a more perfect specimen of small-pox and its varieties than Sydenham offers, or of the gout, or of fevers, than we meet with in the same writer; or of the venereal disease, in all its forms, than Mr. Hunter presents us; or of scarlatina, than Fothergill's; of elephantiasis, than Dr. Adams's; or of bronchitis, than Badman's?—And what assistance have they derived from artificial arrangement? But, it will be said, if we wish to avail ourselves of the labours of others, or to render our own useful, some method is necessary for concentrating the result of these labours. True—then, as we before remarked, nosology is a *catalogue raisonnée* of diseases and of authors; and, if so, we shall leave every writer to chuse his own mode, and the public will judge which is the handiest. For our parts, we have no objection to any, excepting as they endanger decision in the practice of acute diseases, and in chronic or local complaints encourage indolence, by substituting terms for well-defined descriptions. On the influence of nosology in acute diseases, we have already said enough; in chronic complaints, we shall find ample opportunities when we come to notice the various parts of the arrangement before us.

Mr. Good concludes his prolegomena by some remarks on the oriental synonyms and writers. Of the accuracy of these it is not in our power to judge, nor have we, as on all other occasions, consulted those who might give us information. The truth is, we were unacquainted with any medical gentleman who pretended to a critical knowledge of those languages. We felt, indeed, at first, some alarm from learning that "no one ought to pretend to a scientific acquaintance with cutaneous diseases, who has not studied Serapion; nor to a practical history of small-pox, who has not read the pages of Rhazes." Yet it appears, on the same authority, that Willan was unable to read Serapion, and we have no proof that Sydenham knew any thing of Rhazes; yet the honest Boerhaave advised his hearers to read Sydenham, *plus quam decies.* Probably Boerhaave might be ignorant of the treasures contained in Rhazes. Such was not, however, the case with Mead, who procured Latin translations of his work from two different sources, and collated them with the highest authority in his days; yet, after all, he only speaks of Rhazes as containing an imperfect detail of what he knew before, and even demands ample "allowance for time and place." This is very different from his language when speaking of his countryman Sydenham—of whom he remarks that he was the "*first* who divided that disease into different stages, and gave the method of cure in each."

Besides the English translation of Rhazes, by Mead, we have one in Latin, by Mr. Channing, an apothecary of considerable practice in London. This gentleman, after a laborious study, repaired to Oxford, that he might have a more perfect acquaintance with oriental literature, and was so ready at Arabic, that his private memorandums were all made in that language. His translation of Rhazes is from a MS. at Leyden. He has also added the Almanzor, but from from authorities less certain. After the perusal of these translations of the best Arabic works in physic, we feel less regret at our ignorance of that language, especially when we reflect on the first aphorism of the Grecian sage, and that we have heard profound scholars, not destined for medicine, regret the time they had lost in the acquisition of oriental literature. The best part, as far as relates to science, is well known to consist of translations from the Greek. Even Rhâzes, in this celebrated work, would gladly have discovered small-pox in the writings of Galen,—an attempt about as successful as the researches of a modern writer into Hippocrates for a similar purpose.

Whilst we thus submit, perhaps it will be said from necessity, to our ignorance, we feel the more obliged to those who have undertaken a labour from which we have shrunk. Etymology cannot be without its uses, and as Mr. Good has furnished us with the oriental words in their proper characters, we trust, in a future edition, he will imitate Mr. Tooke in furnishing us with alphabets, or an alphabet, with directions how to use it. "Occasionally," we are told, "where the Arabic names are also Persian or Turkish, the author has added the initials, or other marks of these cognate tongues, and, in a few instances in which they are peculiarly expressive, he has also superadded the Persian or Turkish names, even though different from the Arabic. At times, indeed, the Arabic writers themselves employ a Persian or Syriac term, for several of them were of Persian or Syrian birth, and in such cases the author has also indicated the proper origin: all which has been a *labour* of no small *trouble*, from the novelty of the attempt, and the difficulty of procuring medical Arabic and other eastern books that would answer the purpose." It is impossible to doubt the author's diligence, nor can we wonder if, in the midst of such multifarious inquiry into languages, he has occasionally become obscure in his own.

We shall here conclude our remarks on the introductory part of the work. The reader may think them long, but we can assure him they are much shorter than our first MS. The extracts are, indeed, more copious, as we are always fearful of doing injustice to an author by partial quotations, or by offering his opinions in words at all different from his own. Where we differ from him, this is particularly necessary; and, where we agree, there is always danger lest by transferring his thoughts into our own words, we should seem to claim his discovery for our own. It is, however, time time we should enter on the Nosology itself. The following are the

"*Series of Classes and Orders.*

CLASS I.—CŒLIACA—Diseases of the Digestive Function.
Order I.—ENTERICA—Affecting the Alimentary Canal.
II.—SPLANCHNICA—Affecting the Collatitious Viscera."

The six other classes follow, with their orders, after which is a

"*Table of the Affixes and Suffixes that chiefly occur in the ensuing Nomenclature, with the Senses in which they are used.*

| | | |
|---|---|---|
| | AFFIXES. | |
| A | (ἀ) | Diminution or loss of quality or power. |
| Apo, ap, aph | (ἀπο, ἀπ, ἀφ) | For the most part iterative, duplicate, or augmented action: but often indeterminate. |
| Cata, cat | (κατα, κατ) | |
| Dia | (δια) | Separation; secernment; or secretion. |
| Dys | (δυς) | Morbid state or action generally; emphatical, when accompanied with distress or difficulty. |
| Ec, ex | (ἐκ, ἐξ) | Out of; outwards; over; above. |
| Epi, ep, eph | (ἐπι, ἐπ, ἐφ) | |
| Hyper | (ὑπερ) | |
| En | (ἐν) | Within; below: applied to places. Superiority; excess or intensity: applied to quantity or quality. |
| Para | (παρα) | Morbid state or action generally; and hence synonymous with *dys;* except in a few terms derived from anatomy, in which it imports *apud,* 'bordering on,' as in parotitis, paronychia. |
| Peri | (περι) | Circuit; circumference. |
| | SUFFIXES. | |
| Algia | (ἀλγια) | Pain or ache. |
| Asmus, osmus | (ἀσμα, ἀσμος) | Morbid action, power, or possession generally; but mostly very indeterminate. |
| Esmus, ismus | (ἐσμος, ισμος) | |
| Esis, osis | (ἐσις, ωσις) | |
| Iasis | (ιασις) | Cutaneous eruption, unconnected with fever as its cause. |
| Itis | (ιτης) | Organic inflammation. |
| Kele, cele | (κηλη) | Covered protrusion of a soft part. |
| Odes | (ωδης) | Like; a-kin to. |
| Oma | (ωμα) | External protuberance. |
| Ptoma | (πτωμα) | Naked prolapse of a soft part. |
| Rhœa | (ῥοια) | Preternatural flux of any fluid except blood. |
| Rhagia | (ῥαγια) | Preternatural flux of blood. |
| | LATIN. | |
| Igo | | Diffusive or migratory action or motion. |
| Illa, ula | | Simple diminutive terminations. |
| Illaris, ularis | | |
| Osus | | Simple augmentive termination." |

We have transcribed this as a useful table, and which may be readily referred to in reading our future extracts. Only

Only one objection occurs to us, which it will be time enough to notice when we arrive at a passage in which the term is introduced.

In examining the execution of the work, we had determined to proceed *seriatim*. But, as this might prove less interesting to ourselves, and, as we apprehended, to our readers, after a minute attention to the first article, we contented ourselves with those which appeared more connected with nosological controversy, and some of which having come lately before us would be more fresh in our readers' recollection.

"*Genus I.*—ODONTIA. Pain or derangement of the teeth or their sockets.
Odontalgia. *Sauv.*

1. DENTITIONIS. Irritation from cutting the teeth.

Odontiasis. *Paul. Ægin.*

Odontalgia dentitionis. *Sauv.*

Odaxismus. *Vog.*

Ziras (———).

Zahnen. *G.*

Dentition. *F.*

*Teething.*

α Lactántium. Cutting the milk or shedding teeth.

β Puerílis. Cutting the second set or permanent teeth.

γ Adultórum. Cutting the adult or wise teeth.

δ Sen-

---

"Class I. CŒLIACA. Κοιλιακα, 'alvina,' from κοιλία, 'alvus,' 'venter:' and hence the terms cœliac artery, and cœliac passion.

"Order I. ENTERICA. Ἐντερικα, 'intestinalia;' from ἐντερον, 'intestinum,' 'alvus,' 'viscus.'

"Gen. I. ODONTIA. From ὀδους, 'dens.' This word is preferred to odontiasis, first, because the termination *iasis* is now generally indicative of diseases of the skin; and next, because odontiasis has been chiefly limited to a single species of the present genus, o. dolorosa, or *tooth-ache*. In the compounds of ὀδους, odontia is common to the Greek writers, as τριοδοντια, &c."

[Here follows a note concerning the progress of dentition in the temporary and permanent teeth.]

"1. γ *O. dentitionis, Adultorum.*—The cutting of these teeth is often attended with peculiar pain and inconvenience, especially when the process takes place very late, and consequently after the jaw-bones have ceased to grow: for we have in this case often a want of sufficient room, and, in the upper jaw, the tooth on each side is frequently obliged to grow backward, in which position it sometimes presses on the anterior edge of the coronoid process in shutting the mouth, and consequently gives considerable pain. When the same fact takes place in the lower jaw, some part of the tooth continues to lie hid under that process, and covered by the soft

δ Senilium. Cutting teeth in advanced life, or old age.
2. DOLORÓSA. Acute pain in the teeth or their sockets.
Odontalgia. *Hoffm. Lin. Vog. Cull.*

Dentium

---

soft parts, which are always liable to be squeezed between the new tooth and the corresponding one in the upper jaw. Nothing but a free opening will ever suffice in this case, nor even this always; for at times the evil can only be cured by removing the tooth itself.

"1. δ *O. dentionis, Senilium.* Occasionally reproduced as late as at the age of ninety or a hundred. At 92 *Ysabern,* Journ. de Med. tom. xxv. p. 316.—At 100 *Nitzseh,* Ephem. Erudit. Ann. 1666, p. 175.—At 120 *Ephem. Nat. Cur.* Dec. ii. Ann. iii. Obs. 15.

"For the most part, the teeth shoot forth irregularly, and few in number, so as to be of little benefit, and sometimes more injurious than useful, by preventing the approximation of the callous gums, which till now had been employed as a substitute for the teeth. In one instance, though only in one, Mr. J. Hunter informs us, that he was witness to the reproduction of a complete set in both jaws: and he supposes that in all these cases a new alveolar process is formed, as in the preceding sets. 'From this circumstance,' says he, 'and another that sometimes happens to women at this age, it would appear that there is some effort in nature to renew the body at that period.'

"He alludes to a return of menstruation; but there are other facts, and of perhaps a still more singular kind, that point to the same conclusion. The author once attended a lady who cut several straggling teeth at the age of seventy-four, and at the same time recovered her sight so completely as to throw away her spectacles, which she had made use of for twenty years, and to be able to read with ease the smallest print in the newspapers. In another case that occurred to him, a lady of seventy-six cut two molares, and at the same time completely recovered her hearing, after having, for some years, been so deaf as to be obliged to feel the clapper of a small hand-bell, which she always kept by her on a table, in order to know whether she made it ring.

"One of the most singular instances on record is that given by Dr. Slare in the Phil. Trans. vol. xxvii. 1713, as it occurred to his father. At the age of seventy-five, he renewed an incisor lost twenty-five years before; at seventy-seven, he renewed an incisor to supply a similar vacancy; at eighty, all his teeth were hereby rendered perfect; at eighty-two, they all dropped out successively; two years afterwards they were all successively renewed, so that at eighty-five he had an entire new set. His hair simultaneously changed from a white to a dark hue, and his constitution seemed somewhat more healthy and vigorous. He died suddenly at ninety-nine or a hundred.

"2. *Odontia dolorosa.* The varieties are abridged from Cullen, or rather from Sauvages, from whom Cullen has copied them. In the earlier editions of Cullen's Nosology, odontia dolorosa (odontalgia

Dentium dolor. *Cels.* VI. 9.

Zahn-pein. *G.*

Mal

---

gia as he terms it) is arranged as a species of rheumatism after Hoffman. In the fourth and succeeding editions it is raised to the rank of a distinct genus, and placed (xxii) between arthrodynia and podagra. The Anglo-Saxon name for this affection was *toth-ece.*

"There may possibly be other varieties than are here offered. Every tooth has an internal cavity, which commences at the point of its fang, and enlarges as it ascends into its body. This cavity is not cellular, but smooth in its surface; it contains no marrow, but appears to be filled with blood-vessels, which are doubtless accompanied with nerves, which must necessarily be derived from the second and third branches of the fifth pair, though they have never been distinctly traced. In the interior of this cavity the teeth appear to be peculiarly sensible, and hence direct or indirect exposure to the external air, or, in other words, a carious aperture, or a current of sharp air without such aperture, (for the air seems, in many instances, to act through the substance of a sound tooth), will be sufficient to produce acute pain, and is, in fact, the common cause of tooth-ach: on which account, the readiest modes of cure consist in stopping up the aperture with metal or some other substance, defending the tooth from the access of cold, or destroying the nerve by caustics or cauteries through the aperture itself.

"Perhaps the pain called scorbutic may be regarded as an example of the sympathetic variety: that from gout is for the most part a real transfer of action, the organ previously affected being generally at ease, or nearly so, during its continuation.

"For the caries of perfect teeth it is not easy to account. Out of the body they are indestructible, excepting by very powerful chemical agents; and yet, in the judgment of many physiologists, they are nearly in the same state in the body as out of it, being extraneous substances formed complete at first, without vascularity, growth, or internal action, and even destitute of absorbents. Such, at least, was the opinion of Mr. J. Hunter when composing his 'Natural History of the Human Teeth,' an opinion drawn from the impossibility of injecting them, the perfection in which they are produced at first, and their retaining their natural colour after so long a use of madder as a food, that all the other bones of the body have become thoroughly tinged with it. 'But they have most certainly,' says he, 'a living principle, by which means they make part of the body, and are capable of uniting with any part of a living body; and it is to be observed, that affections of the whole body have less influence upon the teeth than upon any other part of the body. Thus in children affected with the rickets, the teeth grow equally well as in health, though all the other bones are much affected; and hence, their teeth being of a larger size in proportion to the other parts, their mouths are protuberant.'

"Admitting the soundness of these experiments, and the accuracy of of this reasoning, it seems impossible that the teeth, when once perfectly produced in the gums, should ever decay: for no action of the living principle can occasion a secretion of those chemical agents which would alone, in such case, be capable of destroying them. It is probable, therefore, that this reasoning is erroneous, and that the teeth are vascular, though the art of injection is incapable of tracing the vascular structure, and the colouring particles of madder-root are not sufficiently attenuate to enter their vessels. Mr. Hunter himself, indeed, appears to speak with some degree of hesitation in the treatise before us; and, in his subsequent treatise 'On the Diseases of Teeth,' offers observations that seem to show he had at that time embraced a different opinion. In the first essay, indeed, he allows that 'the fangs of teeth are liable to swellings, seemingly of the spina ventosa kind, like other bones;' but he immediately adds, that 'there may be a deception here, for the swelling may be an original formation.' Yet, in the second essay, he treats of this swelling as one of the diseases to which the teeth are perpetually liable: he regards the teeth as subject to the common inflammation of other bones, and, like other bones, evincing at times great sensibility through the entire substance of the organ, as well as in the central cavity itself. Nor is it quite certain that the body of a tooth does not occasionally enlarge as well as its fangs; for nothing is more common than for the space produced by extracting one of the grinders of a healthy adult to be filled up by an approximation of the two adjoining teeth. Mr. Hunter, indeed, endeavours to account for this, by supposing that each of these teeth has been pressed into the vacancy by the teeth behind them, in consequence of their want of a proper support in this direction; but, in such case, there must be some vacuity discoverable between themselves and the teeth which have thus urged them forward. In various cases, the author has never been able to trace any such vacuity whatever; and has a decisive example to the contrary in the state of his own teeth: for having, when a boy of twelve years old, had the second of the bicuspidati extracted, the vacancy thereby produced has been so completely filled up by the enlargement of the adjoining teeth, that these teeth closely touch, and he is only able to introduce a fine probe between them at the neck, or lowest and narrowest part; while he can introduce nothing between any of the other teeth, which have in no respect given way or separated from each other.

"There is probably, therefore, some internal action continually taking place, though we are not able to trace it very evidently. And it is probable, also, that a caries of the teeth is occasionally produced by some internal cause operating upon and vitiating this action, though there can be no doubt that the chief causes are external."

α Cariosa. Carious, or from decay.
β Catarrhalis. From cold.
γ Nervorum. Chiefly or altogether confined to the nerves of the sockets or jaw-bone, and not relieved by extracting the suspected teeth. *Hunter* on Teeth, p. 190.
δ Sympathetica. From sympathy: as that of pregnancy, or irritating sordes in the stomach.

Found also, occasionally, as a symptom, in scurvy (porphyra), erratic gout, and hysteric diathesis.

3. STUPÓRIS. Tingling pain in the teeth from stridulous sounds, vellication, or acrid substances.

Hæmodia, (αἱμωδία). *Aristot.*

Odontalgia hæmodia. *Sauv.*

Dolor dentium à stridore. *Darw.*

Zähne-stumpf. *G.*

Agacement des dents. *F.*

*Tooth-edge.*

α A stridóre. From grating sounds.

β Ab acritúdine. From vellication or acrid substances.

4. DEFÓRMIS. Deformity of the teeth from error of shape, position, or number.

5. EDENTULA. Loss or want of teeth.

Nodosia (νωδοσια). *Auct. Græc.*

Nefrendis. *Vog.*

*Toothlessness.*

α Peculiaris. From constitutional defect.

β A vi extrinseca. From external violence.

γ à carie. From decay.

δ Senilium. From old age.

---

[The author here refers to a very general opinion concerning extremes of heat and cold.]

"By whatever means, however, a decay or caries of the teeth may be produced, it appears to operate in three different ways: sometimes commencing in the internal cavity, and working its path outward; sometimes outward, and working its path within; and sometimes by a wasting of the enamel, and consequent denudation of the bony part. The first is the least common affection, and is discoverable by the appearance of the internal blackness through the external shell; the third is more common than the first, and the second the most frequent of the whole; evincing, at its commencement, the appearance of an opake white spot through the enamel, which gradually crumbles away about the spot, and thus discloses that part of the body of the tooth which forms the original seat of the disease, and which, by its continuance, converts the early spot into a hole, and at length destroys it altogether, or at least down to its neck, unless the pain produced by its progress compel the patient to have it extracted before the disease advances thus far."

6. INCRUSTANS.

6. INCRUSTANS. Teeth incrusted with extraneous matter.

*Tartar of the Teeth.* *Hunter*, p. 192.

Concreted by it into one mass. *Eustach.* Tr. de Dent. cap. 2.

7. EXCRESCENS. The substance of the surrounding gums excrescent.

Epulis (ἐπουλὶς). *Paul. Ægin.* iii. 26.

α Spongiosa. Fungous or spungy gums.

*Scurvy of the gums*, vulgarly so called. *J. Hunter*, p. 184.

ϐ Extuberans. With distinct extuberances on the surface.

Epulis. *Heister.* Chir. tom. I. p. ii. c. 85.

Sarcoma epulis. *Sauv.*

Sometimes softer and fleshy. *J. Hunter*, p. 169; and sometimes thicker and callous. *Id.* p. 188. Produced by vermicles."

This description of the cavity in the tooth, and of the three kinds of disease, is similar to Mr. Hunter's. Exposure to cold, is not, we believe, mentioned by him, and it seems too general to be a cause of these diseases. There is something obscure in the manner in which the teeth are said by Mr. Hunter to be vascular and not vascular, with action and without action, making a part of the body and yet being extraneous to it. It should be remembered, that the "Natural History of the Teeth" was the first of Mr. H.'s publications in the year 1771, and that it was sold to the booksellers,—a mode which he was often heard to say he never would repeat. When the second part appeared in 1778, it was tacked to the first, and the whole called a second edition. In 1803, a third edition was published without any alteration, the author being then no more. It is not, therefore, to be wondered if the whole should not seem to harmonize. But a little attention will explain the discrepancy. Mr. Hunter's meaning is, that the teeth when completely formed, do not seem supported like other bones by a circulation from the neighbouring vessels, but, to possess a life of their own, like parasyte animals in a living body. On this account, they have no means of restoration when diseased. Mr. Good is mistaken in saying, that they retain their colour when the other bones are tinged by feeding the animal on madder. If this mode is used for feeding young animals whilst the teeth are forming, they will be tinged; but do not, like the other bones, recover their colour on leaving off the madder, nor can teeth, when completely formed, be tinged by any change of food. It is no argument against their extraneous nature, that they will anchylose with the sockets. Under inflammation, new actions take place, and different parts of different animals may be made to unite, as is well known by Mr. Hunter's experiment of inserting the testicle of a cock into the abdomen of a hen, a hen, and even transplanting a tooth into the comb of a cock; in both which instances blood-vessels are found communicating between the parts in contact. Nor is there any more reason why teeth should not decay because they are extraneous to the neighbouring parts, than that hydatids should not die. But, whether healthy teeth are vascular or not, must be a matter of conjecture till we can discover their vessels. Mr. Hunter has not overlooked the impossibility of the teeth becoming diseased by those menstrua which have a power of destroying part of a tooth, "for any thing of this kind (says he) could not act so partially;"* and it is worth while to mark the coincidence of language between Mr. Hunter and Mr. Good in their account of the various diseases of the teeth, and even in their causes, excepting that the one makes no mention of those external causes, of the existence of which the other has "no doubt." We suspect, however, the events act with too little uniformity to be admitted as of "no doubt." We shall see too, that Mr. Hunter has not been inattentive to all that Mr. Good describes in his own case. In his chapter "Of the Irregularity of the Teeth," Mr. H. shows, that the vacancy from the loss of a tooth may be readily, and often is, filled up by the gradually altered situation of several, which before overlapped each other. He shows also, that the approximation of teeth is most certain early in life. There is, however, one most important disease on which Mr. Hunter bestows much time, but which is not noticed by Mr. Good in this place. It will come before us hereafter in the selection we make of certain articles in the Nosology.

In searching for aneurism, a subject which of late has been often before us, by some error of the index-maker, we were brought the article Elephantiasis.

We were not aware of the length of the present article, till our printer reminded us of several others besides our Intelligence; the remainder must therefore be left for the succeeding Number. Meanwhile, if we have any-where mistaken Mr. Good's meaning, we shall be thankful to be corrected by himself or his friends; for we scarcely know a subject more important than the present, nor of course one on which we are more anxious to be perspicuous and correct.

---

* See Hunter on the Teeth, Part II. Chap. I.

*A Botanical*

*A Botanical Arrangement of British Plants in the Midland Counties, particularly those in the Neighbourhood of Alcester; with occasional Notes and Observations: to which are prefixed, a short Introduction to the Study of Botany, and to the Knowledge of the principal Natural Orders.* By T. PURTON, Surgeon, Alcester. Embellished with Eight Coloured Engravings, by JAMES SOWERBY, F.L.S. Small 8vo. 2 vols. Longman and Co.

THIS is an elegant and very useful compendium, answering all the purposes stated in the title-page, and admirably suited to the female student, as well as to every amateur who wishes for a knowledge of botany rather as an useful accomplishment than a profound study.

---

*Remarks on Arsenic, considered as a Poison and a Medicine; to which are added Five Cases of Recovery from the poisonous Effects of Arsenic. Together with the Tests so successfully employed for detecting the White Metallic Oxide; in which those satisfactory Methods peculiar to Mr. Hume were principally adopted, confirmed, and compared with others formerly in use.* By JOHN MARSHALL, Member of the Royal College of Surgeons in London, and Apothecary to his Royal Highness the Duke of Gloucester's Household, &c. &c. 8vo. pp. 168. Callow, 1817.

OUR readers will probably recollect, that the late Eliza Fenning, and the family of the Turners in Chancery-lane, were visited by Mr. Ogilvie, and afterwards by Mr. Marshall, their family apothecary. This has induced the author to offer some useful remarks on the effect of arsenic as a poison, after a practical experience in so many cases, and also to introduce some further accounts of its effects as a medicine administered in the manner recommended by Dr. Fowler.

The first chapter relates the history of the Turner family, of which we shall offer a short epitome.—The event of the poisoned dumplings occurred Tuesday the 21st of March.

"My attention (says the author) was then directed to Mr. Robert Turner, who appeared to be nearly *in articulo mortis;* his face, which had been swollen, having assumed the appearance of the true *facies hippocratica,* my apprehensions were considerable for his preservation. On examining the contents of the utensils in which he had vomited, a fluid was perceived of a yellowish and greenish colour, and in two of them stercoraceous matter; the pulse was gone, his voice faint and tremulous, and he pointed to the abdomen in great agony. On examination I discovered a very remarkable irregularity of surface, occasioned by the spasmodic contractions of the muscles muscles of the abdomen, and even of the viscera; this unevenness extended from the epigastric region to the *pubis,* and to the right and left *hypochondrium;* and the excruciating pain was relieved for a short time by rubbing the abdomen with a piece of hot flannel and laudanum. From this state of the abdominal surface, there could be no doubt that the arsenic had gone far beyond the limits of the stomach, into the alimentary canal. He complained of extreme faintness, and dreadful sickness. Mr. R. T. had been violently purged; and on examining the alvine secretions, the singularity of their appearance excited great surprise; they were all of a bright homogeneous green colour, like paint, and strongly resembled the green colour produced from a solution of the arsenic by one of Mr. Hume's tests, the ammoniaco sulphate of copper, which will afterwards be more fully described. Each effort of vomiting and purging was preceded and followed by these painful gripings and spasmodic contractions of the abdominal muscles. Mr. R. T. complained of great heat in the stomach, which the patient compared to a furnace, or red hot irons, which sensation commenced at the tongue, and was felt throughout the course of the *œsophagus* to the *cardia,* or upper orifice of the stomach; insatiable thirst, violent head-ach, the eyes impatient of light, but the pupils sensible, and the extremities cold. The patient attempted, in this dreadful state, to get out of bed, to walk to the night table; he was directly seized with vertigo, dimness of sight, and palpitation of the heart; he fell down, and went off into an epileptic fit; he was assisted on the bed, and in a few minutes recovered from the fit.

"Mrs. Robert Turner had great pain and burning heat in the stomach, head-ach, immoderate thirst, vomiting and purging, with olive green alvine discharges, tension of the abdomen, the face swollen, cold chills alternating with flushings of heat; and light was painful to the eyes. Mrs. R. Turner's peculiar situation made me apprehensive of a miscarriage, in consequence of frequent bearing pains more or less constant in the loins; and, independently of these distressing symptoms, her mind was additionally agitated by the alarming state of her husband, who was lying by her side. If Mrs. R. T. had miscarried under these dreadful circumstances, there can be no hesitation in saying, she must have inevitably lost her life.

"I next saw Mr. Turner, senior, with symptoms in many respects similar, though not quite so importunate as in the two foregoing cases: Mr. T. had the burning sensation in the stomach, vomiting, inordinate thirst, head-ach, the face swollen, tension of the abdomen; the purgative symptom had been more moderate. Mr. T. did not complain of light affecting the eyes; and the countenance was flushed, particularly on the upper part of the cheeks."

"Finding Mr. O. had most judiciously and thoroughly emptied and washed their stomachs, and as we had every reason to suspect some portion of the arsenic had escaped into the alimentary canal, especially in the cases of Mr. and Mrs. Robert Turner, and Mr. Gadsden, [Mr. T.'s apprentice,] we resolved to persist in the purgative plan, and gave to each patient another full dose of castor-oil, on two table spoonfuls of milk, and every four hours a solution of the *magnesiæ sulphas* with manna, in mint water; this dose to be alternated every two hours with the saline draught in the state of effervescence, letting the alkali predominate four grains to each dose, with the intention of neutralizing any possible remains of the arsenic, and relieving the disposition to vomit; and we further determined on persisting in the purgative system, until a more natural colour was effected in the alvine secretion. The patients were allowed to drink frequently, and in small quantities, milk, soda-water with or without milk, and mutton broth. Mr. T. and Mr. R. Turner wished to have a draught of porter, but we strongly entreated them, also Mrs. R. T. and Mr. Gadsden, to abstain from beer, wine, and all fermented liquors, which they did for a fortnight afterwards, and likewise from animal food. Mr. R. T. once deviated from these directions, on Saturday the 25th, and the effects will be hereafter described, which will satisfactorily demonstrate the propriety of the plan of diet both here and subsequently recommended. Dr. Orfila, in his admirable Treatise (vide English translation) on Poisons, emphatically confirms this regimen, as in the following extract—'It must never be forgotten, that the success of the treatment depends in a great measure on the sort of regimen the patient observes during his convalescence, which is commonly long and painful. He ought to be principally nourished by milk, gruel, and rice creams, and he should be made to take nourishing broths.' The thirst of each patient was so urgent, that they would readily have drank quarts, had they been permitted; and had we yielded to their request, the vomiting would have been at this time unnecessarily excited, and Mr. O. and myself were apprehensive it might tend to increase the inflammation on the villous coat of the stomach, and augment the symptoms of debility. The thirst was somewhat allayed by frequently washing the mouth with cold water.

"On the following morning, March 22d, I visited the patients, who had all passed a restless night; the vomiting in each had greatly abated, the pain in the stomach was still violent, which they all compared to a furnace, or hot irons; the alvine discharges were changing to a proper colour, but intermixed with streaks of green, and highly offensive; the skin hot and dry, the pulse quick, varying in each case from 100 to 130, great thirst, and violent headach; their tongues white but moist; Mr. and Mrs. Robert Turner, and Mr. Gadsden, could not endure a strong light.

"The supersaturated saline draught *in actu effervescentiæ*, with the addition of manna, was ordered to be continued, and the purgative mixture to be omitted.

"Mrs. R. Turner's pulse was 130; this rapid circulation was accompanied with constant sensations of fainting; but the bearing pains, with the pain in the loins, had somewhat abated.

"Mr. Gadsden appeared this morning to be the most afflicted; he had been seized with four epileptic fits in the course of the night, preceded by a violent palpitation of the heart, accompanied with a peculiar tremulous action of the right arm, and lower extremity; a considerable considerable degree of symptomatic fever, insatiable thirst, a white but moist tongue, the face flushed, the respiration hurried, pulse 126, irregular and contracted, frequent gripings in the bowels, and spasmodic twitchings in the muscles of the chest and abdomen.

"Mr. Robert Turner, in the early part of the morning had another attack of epilepsy; the symptomatic fever ran high, the pulse 120; he complained of spasmodic twitchings about the chest and abdomen, palpitation of the heart, great languor, accompanied with a constant sensation of fainting, tongue white but not dry, occasional chills, followed by an increase of heat, head-ach, and vertigo. A dose of the purgative mixture was administered, and the same medicine as on the preceding day continued.

"Mr. Turner, senior, appeared much better; the pulse 90, skin temperate, tongue moist and cleaner; the vomiting had subsided, but the stomach was in great pain; he complained of extreme lassitude; the face was flushed, and he had slept about four hours.

"The faces of all the four patients were swollen, with a fixed redness, more or less, under the eyes and on the cheek-bones; they had vomited two or three times in the course of the night, by drinking too copious a draught of the diluents recommended over night, and each complained of the tongue and lips being sore and swollen."

Those who wish for minute information, will consult the work; we shall only in general remark, that Mr. Turner and the apprentice had a repetition of epileptic fits, and Mrs. R. Turner was delivered of a healthy child. Mr. T.'s recovery was also retarded by too early a return to a free diet. Mr. Gadsden's epileptic fits have continued to the present time and rendered him unfit for his occupation. The remedies used for the relief of this symptom are slightly mentioned.

The succeeding chapter contains reflexions on the natural and artificial causes which contributed to the recovery of the patients, with practical observations, and some remarks on the treatment of epilepsy. The last, containing a continuation of Mr. Gadsden's case, we shall transcribe.

"Since giving the above description of the state of the constitution and symptoms of Mr. Gadsden, he has returned from Cheltenham, greatly improved in his general health, and was fully able to resume his situation in Mr. Turner's office; the epileptic fits had left him about three weeks; but I have to regret the necessity of stating, that he has experienced a relapse, with increased violence and frequency, as the fits now* return every twelve, or at least three or four times in forty-eight, hours. For some minutes his sensations and distress of countenance generally indicate their approach; and since the short time the epilepsy has returned, the patient's health and strength have suffered materially.

"In epilepsy, phlebotomy is almost generally recommended;

"* About three months subsequent to the commencement of the attack."

 perhaps perhaps it may be of use in some plethoric cases, wherein the blood is liable to determine towards the vessels of the head, and in those patients who are more advanced in years or verging upon a state of apoplexy, or when attributable to the sudden suppression of a customary discharge, especially the catamenia. But in the course of my practice I have never witnessed any material advantage derived from bleeding in this disease. After the second relapse of the epileptic fits, Mr. G. was bled by the advice of Dr. Babington, but without the slightest mitigation of the violence or frequency of the attack; he was, therefore, again recommended to change the air, from which he derived considerable benefit. Two years have now elapsed since Mr. G. became thus afflicted by the arsenic, and he is still subject to frequent and severe recurrences of this frightful disease."

Some remarks follow on the use of ol. terebinth. in epilepsy, with the authority of Drs. Penderleath and Young, and of Drs. Merriman and Latham in worms, which the author considers a frequent cause of epilepsy.

In considering arsenic as a medicine, particularly in the form of Fowler's solution, the author informs us of ill effects which, after a very extensive practice, have never occurred to us. We much suspect sufficient care was not taken, that this powerful medicine should never be applied to the naked stomach. As a remedy, Mr. Marshall recommends magnesia. We are not prepared to say what the effects of this substance may be in cases in which arsenic has proved deleterious; but, from the facility with which we have for many years exhibited Fowler's solution, we should be unwilling to introduce any other, or to render that more complicated.

Having given this gentleman's account of these cases, and of the author's and our own opinion of arsenic as a remedy, we shall conclude with remarks on the test by which it is to be discovered when swallowed.

Of all the tests discovered for proving the existence of arsenic, our author gives a decided preference to those peculiar to Mr. Hume; and, amongst these, he quotes that which first appeared in our Journal, (Medical and Physical Journal, 1810). This consists in converting the white arsenic into arseniate of potash, and may be adopted either primarily to detect the poison, or subsequently to the silver-test, to substantiate the truth where there is any doubt. The results of some experiments on *decoction of onions* are detailed, and these seem to invalidate the opinions of some medical men, on a late trial in Cornwall, who affirmed, that this decoction may be confounded with a solution of arsenic. Besides the author's own testimony, we have that of Dr. Plenderleath and Mr. Hume, all agreeing that there is no similarity between these fluids to warrant such assertions, or to depreciate depreciate the character of these tests. There are some very useful remarks upon the operation of these reagents with *phosphates*, and cautions to operators to avoid false conclusions, in their progress through an enquiry of such consequence to the welfare and safety of society. We are pleased to find, that the test with nitrate of potass may be so modified as to distinguish at once the difference between arsenite of silver and a phosphate of the same metal. This method is fully described at page 159, and deserves the best attention of our readers, especially of those who are conversant in chemical investigation.

---

*Edinburgh Medical and Surgical Journal*, No. LI. July, 1817.

(*Continued from p.* 155.)

Art. V.—*Case of Wounded Bladder, terminating favourably.* By J. Douglas, Surgeon, Hawick.

This paper contains much interesting and useful information, conveyed with such brevity that we may hereafter be induced to transcribe it.

Art. VI.—*History of a Case of Dislocation of the Lower Jaw; with Remarks on the Sentence of a Court Martial held to investigate the Nature of the Causes that produced it.* By John Forbes, Esq. Surgeon, Royal Navy.

This paper contains matter of too delicate a nature for us to enter on without a more complete knowledge of all the events connected with the parties.

Art. VIII.—*Observations upon Diseased Spine, with a Case.* By J. B. Estlin, Surgeon, Bristol.

The first part of this paper contains a general analysis of Mr. Baynton's Essay, and of Mr. Earle's remarks on the same. Some incidental observations follow in favour of Mr. Copeland's work on the same subject. In one part, Mr. Estlin seems to express a surprise that Mr. Baynton has not thought proper to reply to Mr. Earle. In our opinion, Mr. B. has acted wisely, for, though there cannot be any question of Mr. Earle's abilities, or of the good sense contained in his paper, yet the difference of opinion is not such as to require explanation. The facts are sufficiently before the medical public to enable them to form a proper estimate. We acknowledge, at the same time, that, from long experience in that school in which the caustic practice, if it did not originate, was carried to the greatest extent, we have often felt doubts of its efficacy; and, even in Mr. Estlin's case, we should have preferred the free use of the cupping glasses to the seatons or issues. From experience, we do not scruple to recommend topical bleeding in similar cases, as a much quicker, pleasanter, and more certain remedy. We own ourselves somewhat at a loss that surgeons of such eminence as were consulted before Mr. Estlin should have advised nothing but the horizontal posture for a disease of so long continuance, and which they considered as inflammation going on in the back. The case is, indeed, so similar to some with which we have lately met, that we might almost transcribe it as one of our own. The following is Mr. Estlin's:

"In February, 1811, Miss ——, aged 26, after a ride on horseback of about twenty miles, felt, for the first time, a weakness in the back, and a general sense of languor; to remove which, she was advised to lie down upon her back during a part of every day. She followed this advice, and, in a few weeks, felt no more of the indisposition. In the following winter, she used great exertion, by taking frequent and long walks, for nearly four months, and again felt a local weakness in the back: there was also, she recollects, some degree of tenderness in the vertebræ when pressed. These symptoms were also relieved by occasional recumbency and repose from much exercise; but, during the summer (1812), the uneasiness returned so constantly, that she was obliged to lie down the greatest part of the day. She always felt easy while lying down. In September, by the advice of a medical gentleman, she put on a machine, which was constructed to remove the weight from the spine, and throw it upon the pelvis. During the next twelve months, she either lay down, or sat with the assistance of this machine: she used to sit up for one hour, and lie down two or three; then sit up for an hour, and so on. At the expiration of these twelve months, she was, upon the whole, worse: the sensation in the back, which she has uniformly described as more a feeling of weakness than of actual pain, had increased; talking, laughing, or crying, even while lying down, always produced a heat in the back; and she constantly felt a sensation as if bits of horse hair were pricking the back. At this period, September 1814, Mr. Cline was consulted by letter: he said, that he considered the symptoms those of inflammation going on in the back, and recommended rest in a horizontal posture. Another eminent surgeon, residing at a distance, was also consulted by letter, who thought the case was one of diseased spine, and recommended, as Mr. Cline had done, undeviating rest in a horizontal posture. This plan was scrupulously persevered in from September 1814, to April 1815. The patient lay, for the first two months, upon a hair-mattress, but, fearing it might be too yielding, she had it removed as far as the small of the back, and a board, covered with blankets, upon a level with the upper part of the mattress, was substituted.

"On the 14th of April, 1815, I saw her for the first time since her indisposition. My notes say, that she feels exactly in the same state state in which she has been for some months. When perfectly at rest, and not speaking, she experiences no inconvenience. Moving the arms, and talking, affect her most. Yawning, sneezing, laughing, crying, and coughing, occasion the sensation of weakness and heat between the shoulders, or, as she expresses it, 'under the lungs.' She describes the general feeling as 'a want of compactness in the vertebræ.' If she hold up a book for a short time only, her back becomes heated, and she has slight pains, 'like lines as small as horse hairs, and about an inch in length.' To communicate an idea of her feelings, she also describes them to be 'as if all the ligaments of the body were suspended by a wooden peg between the shoulders.' An obstinately constipated state of the bowels exists. During the day, her couch is wheeled into an airy drawing-room; and her clothes are contrived so as to be put on and taken off without her removing her back from the mattress. When she feels the uneasiness in the back most, she is able to refer it to a particular and small spot between the shoulders.

"Upon examining the spine attentively, no distortion or curvature was perceptible. Pressure with the fingers upon the cervical vertebræ occasioned no uneasiness, but, when it was applied to the third or fourth dorsal, she started suddenly away, and said her breathing was impeded by it. The sensation thus produced, she said, could not properly be called pain. The same tenderness existed in several of the succeeding bones, but the principal seat of it was between the third and seventh or eighth. There was not the least tenderness in the lumbar vertebræ.

"After this examination, I could entertain no doubt that several of the dorsal vertebræ were affected by disease; and, when I considered how long the patient had been indisposed, and how slight a degree of pressure occasioned the sudden starting and uneasiness she complained of, I was led to fear that the progress of the inflammation had been considerable, though no curvature or paralysis had yet appeared. The custom she had so long adopted of lying down whenever the back was uneasy, and of thus relieving pressure, was probably the cause of the complaint's not having manifested any appearance of distortion or palsy.

"As the plan of undeviating rest had been persevered in for seven months, without producing any considerable amendment (though she thinks she felt a little benefit from it,) I did not hesitate to propose the immediate establishment of issues on each side of the affected part of the spine, and could not help feeling regret, with my views of this disorder, that so much time had been suffered to elapse without their having been had recourse to. I had, however, the pleasure of finding that the result did not disappoint my most sanguine anticipations.

"I applied the potassa fusa to a circular space, of about an inch and a quarter in diameter, on each side of the tender vertebræ. She lay upon her face for about twenty minutes while the eschars were forming. The surrounding skin became considerably inflamed. On the evening of the day upon which the caustics were applied, she said said her back felt less uneasy than it had been for some time before, though she had used more exertion than she had done for a considerable preceding period.

"As the lady who is the subject of this case lived at a great distance from Bristol, I was obliged to leave her the day after the application of the caustics; but she was fortunate enough to remain under the occasional observation of a gentleman who is eminent for his professional abilities, to whose judicious co-operation I feel much indebted, as instrumental to the happy termination of a case, where feelings of private friendship added to the interest which, in a professional point of view, I naturally took in it.

"When the eschars formed by the caustic had sloughed away, the openings were kept discharging by the insertion of a large Windsor bean into each; and, on the 15th of May, a month after the caustics had been applied, I received the following account:—

"'She has not felt lately the heat and irritation she used to have in the back, excepting one day when she coughed a great deal and sneezed. She turns upon her side more easily than she did. Talking does not produce so much local heat as it did.'

"June 11.—'The vertebræ are as tender as they were at first, but the catching of the breath when they are pressed by no means so great. The issues discharge freely.'

"I cannot give a better idea of the immediate good effects of the issues, than by transcribing her own words from an account of her case which she has since favoured me with. Speaking of her state previous to the period of my seeing her, she says,—

"'From September 1814, to April 1815, I simply lay down, and certainly felt benefit, but still the inflammation in the part continued on the smallest exertion; even the weight of my hands crossed upon my chest was a burthen; and the least movement of hand or foot seemed to bear on the weak part only. Coughing or sneezing I dreaded, as the jerk seemed to strain the part. In April you applied the caustics; from that time I felt nothing of the pricking: the effect seemed instantaneous.'

"The symptoms gradually abated. The caustic was applied to a fresh part, and the former openings suffered to close. In all, five issues were made. The fear of producing any recurrence of the symptoms by a premature remission of the plan that had proved so beneficial, was the cause of its being persevered in longer, perhaps, than was absolutely necessary. In February 1816, as no uneasiness was felt upon pressing or knocking the vertebræ, and the catching of the breath having ceased for some months before, on the application of pressure, she began to be raised during the day a little from the horizontal posture, by means of a contrivance in the crib for the purpose. There was a hinge at the part where the bottom of the back came, which enabled the upper part of the trunk to be raised towards the sitting posture. Soon after this, her progress received a little check, owing to a catarrhal attack, attended with cough; and frequently, during her confinement, she had to contend with the depressing influence of mental anxieties. It is not, then, to be wondered dered at that she did not venture to sit upright before the December following. On the first day of the present year she stood, and in a fortnight after was able to walk. In the latter end of last March I saw her again, and examined the back. There was no more sensation upon pressing the vertebræ than the soreness of the skin produced by the issues, which were then inflamed, fully accounted for. Her amendment has continued. The issues have been suffered to heal. She goes up and down stairs, walks out, and is able to indulge in the recreations of playing, singing, and drawing. Her back is free from uneasiness, and her general health unimpaired. It may not be amiss to observe, that the 'suffering' occasioned by the issues was too trifling to be named."

From reiterated experience we do not scruple to recommend the frequent loss of blood by cupping near to the parts affected. In one of our cases, the patient was not able to recline for a considerable part of the day; and in another, from her rank in life, a convenient couch could not be contrived. In this last, the distortion was considerable; yet both recovered in the course of a few months.

*Cases of Catarrhus Hepaticus;* by JOHN HAY, Esq. Surgeon, 2d Battalion Hon. E. I. C. Artillery, Madras.

We often hear of "great cry and little wool;" of this paper we should say—Much Latin and little science or information. On the whole, however, we cannot conclude these remarks without expressing our satisfaction at the copiousness and respectability of the communications contained in this number.

---

