# Errata

**Published:** 1817-10

**Authors:** 


*Errata in this Article in our last Number.*

The hurry with which it was printed, and the numerous alterations made by the writer, must apologise for the following errata:

Page 218, l. 9 from the bottom, for *á* read *a*.
219, for *morbus* read *morbis*.
219, near the bottom, dele *Dr.* or add it to the other names.
228, for *synopsis* read *synopses*.
239, l. 9 from bottom, add *to* after *brought*.
224, *note*, the reader will correct the quotation from Hippocrates or Mead.

